# Participatory Surveillance of COVID-19 in Lesotho via Weekly Calls: Protocol for Cell Phone Data Collection

**DOI:** 10.2196/31236

**Published:** 2021-09-27

**Authors:** Abigail R Greenleaf, Gerald Mwima, Molibeli Lethoko, Martha Conkling, George Keefer, Christiana Chang, Natasha McLeod, Haruka Maruyama, Qixuan Chen, Shannon M Farley, Andrea Low

**Affiliations:** 1 ICAP at Columbia University Mailman School of Public Health Columbia University New York, NY United States; 2 Department of Population & Family Health Mailman School of Public Health Columbia University New York, NY United States; 3 ICAP at Columbia University - Lesotho Mailman School of Public Health Columbia University Maseru Lesotho; 4 Division of Global HIV/AIDS Center for Global Health US Centers for Disease Control and Prevention Atlanta, GA United States; 5 ICAP at Columbia University - Tanzania Mailman School of Public Health Columbia University Dar es Salaam United Republic of Tanzania; 6 Department of Biostatistics Mailman School of Public Health Columbia University New York, NY United States; 7 Department of Epidemiology Mailman School of Public Health Columbia University New York, NY United States

**Keywords:** COVID-19, cell phones, mHealth, Africa south of the Sahara, surveillance

## Abstract

**Background:**

The increase in cell phone ownership in low- and middle-income countries (LMIC) has created an opportunity for low-cost, rapid data collection by calling participants on their cell phones. Cell phones can be mobilized for a myriad of data collection purposes, including surveillance. In LMIC, cell phone–based surveillance has been used to track Ebola, measles, acute flaccid paralysis, and diarrheal disease, as well as noncommunicable diseases. Phone-based surveillance in LMIC is a particularly pertinent, burgeoning approach in the context of the COVID-19 pandemic. Participatory surveillance via cell phone could allow governments to assess burden of disease and complements existing surveillance systems.

**Objective:**

We describe the protocol for the LeCellPHIA (Lesotho Cell Phone PHIA) project, a cell phone surveillance system that collects weekly population-based data on influenza-like illness (ILI) in Lesotho by calling a representative sample of a recent face-to-face survey.

**Methods:**

We established a phone-based surveillance system to collect ILI symptoms from approximately 1700 participants who had participated in a recent face-to-face survey in Lesotho, the Population-based HIV Impact Assessment (PHIA) Survey. Of the 15,267 PHIA participants who were over 18 years old, 11,975 (78.44%) consented to future research and provided a valid phone number. We followed the PHIA sample design and included 342 primary sampling units from 10 districts. We randomly selected 5 households from each primary sampling unit that had an eligible participant and sampled 1 person per household. We oversampled the elderly, as they are more likely to be affected by COVID-19. A 3-day Zoom training was conducted in June 2020 to train LeCellPHIA interviewers.

**Results:**

The surveillance system launched July 1, 2020, beginning with a 2-week enrollment period followed by weekly calls that will continue until September 30, 2022. Of the 11,975 phone numbers that were in the sample frame, 3020 were sampled, and 1778 were enrolled.

**Conclusions:**

The surveillance system will track COVID-19 in a resource-limited setting. The novel approach of a weekly cell phone–based surveillance system can be used to track other health outcomes, and this protocol provides information about how to implement such a system.

**International Registered Report Identifier (IRRID):**

DERR1-10.2196/31236

## Introduction

The proliferation of cell phone ownership in sub-Saharan Africa (SSA) [1-3] over the past 10 years has created the opportunity to collect health data via cell phones [4-6]. As of 2019, 45% of the population had mobile services, and 50% of the population will have a phone by 2025, translating to almost 1 billion people in SSA owning a cell phone [7]. Because cell phones have created an opportunity for low-cost, rapid-data collection, public health actors use cell phones for a myriad of public health purposes, creating a growing evidence base about the feasibility and validity of cell phone surveys [8-10].

In SSA, the main remote data collection modes are interactive voice response (IVR — “automated voice calls”), SMS, and computer-assisted telephone interviews (CATI — live interviewer administering the survey). Because literacy is not universal in many low- and middle-income countries (LMIC), CATI — although usually the most expensive approach — is the ideal mode for cell phone surveys aiming to contact the general population. When contacting a known population such as health professionals, who are literate and should have digital proficiency, IVR or SMS can be appropriate. Response rates are significantly higher using CATI than using IVR or SMS [4]. Although response rates vary by country, a recent study in Nigeria documented a 15% CATI random digit dial (RDD) response rate, 3% for IVR, and 0.2% for SMS [11]. RDD is a popular sampling approach [10,12], but even with quota sampling, RDD consistently creates a sample that is more male, educated, and urban than the target population [2,11-14]. Therefore, the optimal way to interview a representative sample by cell phone is to enroll participants during a face-to-face interview [13,15].

A critical application of remote data collection in global health is surveillance. Surveillance by phone is low-cost, is efficient, and allows data collection in remote locations. Surveillance via cell phone can be conducted using any of the aforementioned modes to collect data from either health facility staff, community health workers, or lay people. Studies in the Central African Republic [16] and Togo are examples of health care workers using apps to increase the completeness and timeliness of disease surveillance reports coming from health facilities [17]. In Côte D’Ivoire, a surveillance system relied on community health workers to text health facility staff if any of 5 infectious diseases (suspect measles, yellow fever, acute flaccid paralysis, cholera, and meningitis) were detected. This approach resulted in the first 3 of the aforementioned diseases having substantially higher reporting than before the system was implemented [18]. In a similar design to the study in Côte D’Ivoire, health care workers in Niger participated in a human (acute flaccid paralysis and measles) and animal (rabies and peste des petits ruminants) surveillance system via weekly calls [19].

In contrast, participatory surveillance engages a public (lay) population at risk to report on their health-using technology (often using a mobile phone via internet, SMS, or calls) to collect data independent of the health care system [20]. In Tanzania, women in an informal settlement were enrolled via convenience sampling and randomized to send either daily or every-other-day text messages about occurrence of their child’s diarrhea [21]. Over the 4-month study, the overall response rate for the study was 47%, and diarrhea was reported more frequently during daily texting compared with less frequent texting. Although examples of participatory surveillance in SSA are limited, participatory surveillance has increased worldwide due to the COVID-19 pandemic [22-24].

The increased use of cell phone–based surveillance is pertinent in the context of the COVID-19 pandemic since in-person data collection was discouraged, particularly at the beginning of the pandemic. All countries have faced substantial challenges confronting the COVID-19 pandemic. LMIC, in particular, were able to address certain challenges such as improving lab capacity quickly, but other challenges posed by COVID-19 such as underdeveloped surveillance systems require long-term investment to improve [25]. Thus, participatory COVID-19 surveillance can be valuable in shaping the national response and allocation of resources. Participatory surveillance data, which would allow governments to assess COVID-19 burden, mortality, and location of outbreaks, would complement existing surveillance systems and be shared with country COVID-19 task forces who would respond to new cases following national response guidelines.

The cell phone–based participatory surveillance system presented in this manuscript was enacted in Lesotho, a landlocked country with a population just over 2 million people within Southern Africa. After a lockdown from April 30, 2020 to May 5, 2020, Lesotho reported its first case of COVID-19 on May 13, 2020 [26]. This is in contrast with neighboring South Africa, which by the end of April 2020 already had over 5000 cases [27]. The difference between countries can mostly be attributed to a lack of testing in Lesotho, as the country relied on testing suspect cases in South Africa, where the infrastructure was overwhelmed. COVID-19 infections in Lesotho spiked in December 2020 due to the influx of migrant workers returning from South Africa for the holiday season [28]. As of April 2021, Lesotho had conducted 71,129 COVID-19 tests, of which 10,707 (15.1%) were positive [29], and recorded 315 deaths [30].

The participatory surveillance system we present (called LeCellPHIA [Lesotho Cell Phone PHIA]) fulfills the first aim of the World Health Organization’s global COVID-19 surveillance objectives as published on March 20, 2020: monitor trends in COVID-19 disease at national and global levels [31]. Specifically, we tracked influenza-like illness (ILI) by conducting surveillance via a cell phone survey targeting recent face-to-face survey participants to create a population-based, nationally representative estimate of COVID-19 disease burden in Lesotho. The objective of this manuscript is to detail the methods used to implement the participatory surveillance system.

## Methods

### Study Design

#### Overview of Study Design

We are contacting participants via CATI weekly for 27 months (July 1, 2020 to September 30, 2022) to report ILI symptoms as a proxy for COVID-19 symptoms. We inquire about fever, dry cough, and shortness of breath for the participants as well as their household members. As this is syndromic surveillance, we do not ask about testing.

#### Parent Study

With an HIV prevalence rate of 25.6%, Lesotho has one of the highest rates in the world [32]. The 2020 Lesotho Population HIV Impact Assessment (LePHIA2020) was a cross-sectional, household-based, nationally representative survey that assessed the prevalence of key HIV-related health indicators such as HIV incidence, prevalence, viral load suppression, and risk behaviors and described uptake of key HIV prevention, care, and treatment services [33]. This 2-stage cluster survey took place between December 2019 and March 2020 and included 9665 households and 16,466 individuals, with a 93.1% household response rate. All adults aged 15 years and older in the household who slept in the house the night before were invited to participate. There was a 93.6% individual interview response rate and 93.2% HIV testing response rate.

#### Sample Frame

**Figure 1:**
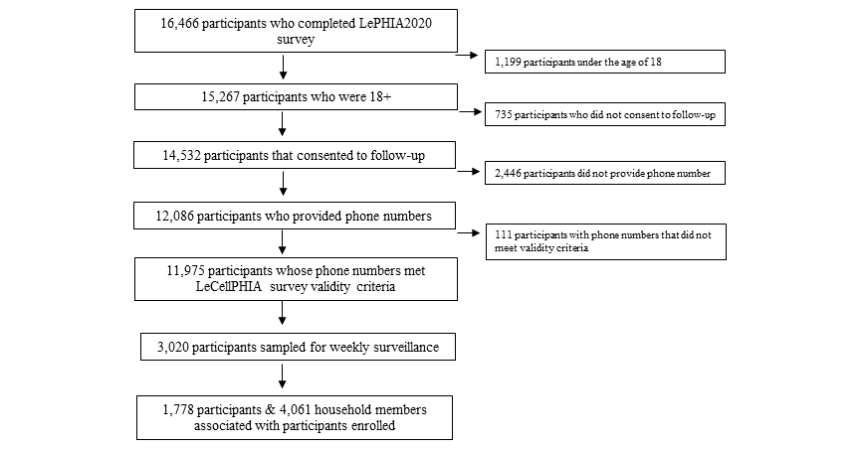
Study enrollment flowchart. LeCellPHIA: Lesotho Cell Phone PHIA; LePHIA2020: 2020 Lesotho Population-Based HIV Impact Assessment; PHIA: Population-based HIV Impact Assessment.

All LePHIA2020 participants who completed the survey were asked if they agreed to be contacted for future research in the next 3 years. The sample frame included all LePHIA2020 participants aged ≥18 years that completed the LePHIA2020 interview, consented to follow-up, and provided a valid phone number. This equates to approximately 11,975 participants of the 15,267 people aged 18 years and older (79%) who were interviewed for LePHIA2020 (see Figure 1).

#### Cleaning Phone Numbers

The LePHIA2020 data are collected via tablets which allows for data quality assurance. The sole data entry parameter for the question about the participant’s phone number was that the data had to be numeric. Therefore, phone numbers of varying lengths were entered, of which some included country codes. With the goal of creating a sample frame that includes only valid phone numbers, a data analyst worked in close collaboration with the Lesotho team to identify phone numbers that were numerically feasible. For this study, phone numbers between the length of 8 and 12 digits — but excluding 10 digits — were considered valid. This range allowed for Lesotho and South African phone numbers with and without a country code. Phone numbers with a length of 10 digits are invalid because no definite rule can be given to determine whether it was a South African number with a missing digit or a Lesotho number with an extra digit. Depending on the number of the digits the phone number started with, it was classified as a Lesotho or South African phone number; then, all phone numbers were coded in a consistent manner so that the interviewer could copy and paste the phone number from the survey software into the phone.

#### Pulsing

Despite including only numerically feasible phone numbers in our sample frame, we still expected a notable amount of nonresponse. To improve the estimate of the percent of phone numbers that would be classified as noncontact, we conducted “pulsing” [34]. Before creating the sample, 3 supervisors called 100 phone numbers that were randomly sampled and excluded from the LeCellPHIA sample frame. Pulsing is calling each phone number once, and if the call rings, allowing the call to ring only once before hanging up. The goal was not to speak to the person whose phone number we called but to record whether the phone number had the possibility of being answered. There were 3 possible outcomes for each call: the call rang; the subscriber was unavailable (due to network issues, the participant being out of service, or the phone being switched off); or the phone number did not exist, which meant the phone number was no longer in use and thus could not be answered.

#### Sampling

For LeCellPHIA, we followed the sample design for LePHIA2020 by including all 342 primary sampling units (PSUs) from the 10 districts. Within each PSU, we oversampled households (HHs) with elderly defined as age ≥60 years, with sampling ratio of 2:1 between HHs with and without elderly. We randomly selected 5 HHs in each PSU from those HHs that had eligible participants. From each sampled HH, 1 person 18 years of age or older was sampled. Based on prior cell phone studies in SSA, we assumed a 25% noncontact rate and 10% refusal rate. To obtain the target sample size of 5 persons per PSU (1710 persons across all PSUs), we called approximately 9 persons in each PSU in the first call (3020 persons across all PSUs) to end with approximately 1710 people in our sample. To increase our sample size, we asked all 1710 people about their household members’ symptoms (average of 2.9 people per household). Given that the virus can spread among family members, we set an intraclass correlation (ICC) of 0.25. Assuming an ICC of 0.25 and average HH size of 3, the design effect within the household was 1+(3[average size of HH]– 1)*0.25 [ICC]= 1.5. Thus, the effective sample size in each HH was 3/1.5 = 2, resulting in an effective overall sample size of 3420.

#### Training

All interviewers were recruited from the recently finished LePHIA2020 and thus had been previously trained by ICAP in ethics, building rapport, and using tablets for data collection, among other topics. Survey personnel were selected based on their LePHIA2020 performance, and all were proficient in English and Sesotho. Before training, each interviewer received a tablet, headphones, Wi-Fi router, and a lockbox to secure the aforementioned equipment. The 2 supervisors underwent a 1-day training followed by a 3-day LeCellPHIA interviewers’ virtual training via Zoom. All interviewers connected from their homes. Staff in the Lesotho ICAP office joined the meeting in a socially distanced room. The curriculum of the training covered how to conduct phone interviews, how to use the software (SurveyCTO), objectives of the research, workflow, responsibilities, monitoring, and COVID-19 information. Specific to COVID-19, survey staff were trained on COVID-19 transmission risk factors, how to mitigate spread in the home and in the community, and other general knowledge about the virus. Slack, a channel-based message platform, is used as the main communication channel between interviewers, supervisors, and ICAP staff. There are Slack channels to communicate about process-related challenges and COVID-19 questions, and there is a private channel for interviewers to communicate and a private channel for supervisory-level staff.

#### Pilot

All 20 interviewers practiced the survey by calling both LeCellPHIA supervisors and 20 randomly selected LePHIA2020 participants who consented to follow-up but were not part of the selected LeCellPHIA sample. Each interviewer made at least 15 calls; these were recorded and reviewed by supervisors and the survey coordinator to evaluate how each interviewer performed. We used the pilot performance to select the 16 best-performing interviewers for the survey, while 4 remained on a hiring wait list.

#### Questionnaire

**Figure 2:**
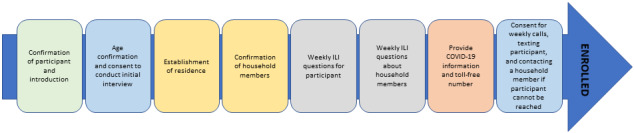
Lesotho Cell Phone Population-based HIV Impact Assessment (LeCellPHIA) participant enrollment steps. ILI: influenza-like illness.

**Figure 3:**
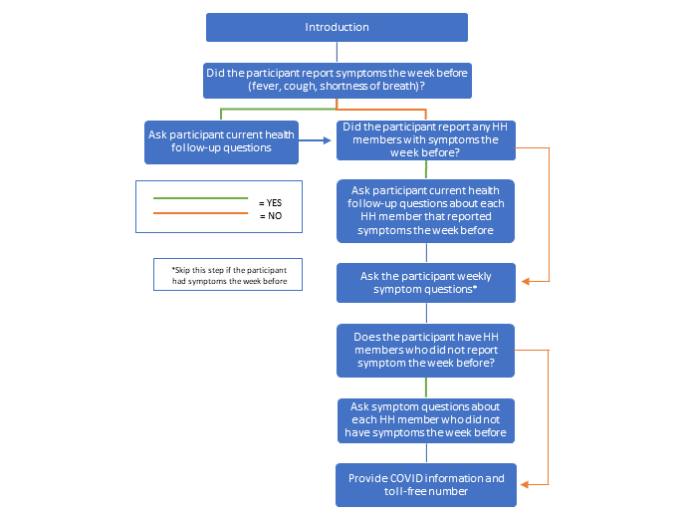
Weekly surveillance questions for Lesotho Cell Phone Population-based HIV Impact Assessment (LeCellPHIA) participants about influenza-like symptoms experienced by themselves and household (HH) members.

The participatory COVID-19 syndromic surveillance questions were developed using Centers for Disease Control and Prevention (CDC) guidance and through consultation with local staff. The enrollment questionnaire, administered only during the first 2 weeks of data collection, had 28 questions (Figure 2). The weekly surveillance questionnaire has between 8 and 11 questions, depending on whether the participant or household member is reported sick the previous week (Figure 3). Abbreviated verbal consent scripts, questionnaires, and other participant-facing documents were translated by ICAP into Sesotho from English.

#### Software

Questionnaires were programmed into the SurveyCTO format using a Microsoft Excel template, then uploaded onto the project’s SurveyCTO server. SurveyCTO’s case management system allows the data management team to assign interviewers a weekly participant list to call. Each week, the data management team generates a new list based on the previous week’s results.

Using the SurveyCTO Collect version 2.70.3 mobile application on an Android (operating system 7) tablet, interviewers access the participant list assigned to them to conduct their weekly interviews. The participant list automatically updates (ie, removes a participant) when a submitted form indicates any of the following: (1) a participant completed the interview for the week or (2) a participant withdrew from the study. If a participant is called 4 times in a week (and a form was submitted each time), then the participant is also removed from the list as 4 is the maximum number of contacts in a week. Once a form is saved and finalized on the SurveyCTO Collect app, it is encrypted, and the data are no longer accessible on the device. Once the data are sent to the SurveyCTO server, the data can only be downloaded using a private encryption key.

SurveyCTO’s audio audit feature is used for supervision purposes. A random selection of 20% of calls is recorded for supervisors to listen to. Due to security features on the Android operating system of the devices used for data collection, only the interviewer side of the call could be heard.

#### Enrollment

To enroll a random sample of approximately 1710 participants, 16 interviewers were assigned 150 participants to call. Interviewers called participants from a private space in their own home or office, ensuring confidentiality. The interviewer wore headphones with a microphone to improve the acoustics and privacy of the calls. The interviewers enrolled participants between July 1, 2020 and July 13, 2020. If the phone number was busy or not picked up, interviewers called back later for a minimum of 7 call attempts over the 2 weeks, using alternate phone numbers if provided. During the enrollment period, there was a daily debrief call between the afternoon and evening shift for all staff that had worked that day. Interviewers called approximately 20 new participants per shift for 7 shifts, then used the remaining 3 shifts at the end of enrollment to solely call back noncontacts.

Participants who answered the phone call were eligible if they confirmed they participated in LePHIA2020, lived in the same home where LePHIA2020 was conducted or planned to go back to the home in the next year, confirmed they were over 18 years of age, provided abbreviated verbal consent, and could hear and understand questions in English or Sesotho. Participants who were otherwise eligible but were not currently living in the house where they answered LePHIA2020 questions were put on a monthly callback list if the participant indicated they would return home sometime in the next 12 months.

During the enrollment phone calls, interviewers oriented the participant to the purpose of the call, confirmed eligibility, and if eligible, administered up to 3 consents. The initial consent included information about the purpose of the survey, described the requirement of the participants, clarified that participation is voluntary, outlined the anticipated burden (length of study), and provided information on the incentive. Once consented, the interviewer then used the household roster from LePHIA2020 to establish which household members were still living with that participant and asked about ILI symptoms for the participant and their household members. The second abbreviated verbal consent, which occurred at the end of the baseline phone call, consented the participant to weekly calls to ask about ILI symptoms for the participant and their household members. If the participant consented, a third abbreviated verbal consent asked if the participant agreed for the interviewer to contact a household member who provided their cell phone number during LePHIA2020 or to SMS the participant, in case we could not get ahold of the participant for 2 or more weeks.

#### Procedures and Weekly Surveillance

After 2 weeks of participant enrollment, weekly surveillance began. A data collection week begins on Thursday and ends on Tuesday. Every Sunday evening, the New York team produces a list of participants who have yet to be called during the past survey week to help ensure that all study participants are called at least once by the end of collection week. Calling does not occur on Wednesdays so as to give the data team time to create new call lists for interviewers based on the previous week’s results (for example, removing a participant who refused or changing the questions for a participant who reported being ill).

If a participant or their household members report ILI symptoms the week before, the participant is first asked if those symptoms have improved. Then, interviewers inquire about participants and their household members who did not have ILI symptoms the week before. All participants are asked if they would like the Government of Lesotho’s toll free COVID-19 hotline phone number. Interviewers offer to answer participants’ questions about COVID-19 at the end of each call.

Interviewers conduct calls mornings, afternoons, evenings, and weekends to increase the likelihood of contacting participants. Interviews are scheduled at specific times that potential participants designated, according to their schedules. Interviewers call the same participant for the duration of the study. For the first month of data collection, interviews took place 7 days a week. After reviewing the data for patterns in responsiveness, the team decided that no day had a particularly high response rate, so the staff started working 6 days a week and eventually 5. As the survey continued, the staff adjusted the days and times that they work to 4 shifts a week.

If a participant cannot be contacted for 2 weeks and they consented during enrollment for the interviewer to call a household member, the household member is called. If a participant cannot be contacted for 2 weeks and they had not consented to contacting a household member but consented to receive a text message, the interviewer texts the participant. If a participant who had consented to contacting a household member is not reached via the household member for a week, the participant is texted the following week by the interviewer if the participant consented to being texted.

If a participant cannot be contacted for 2 months, they are removed from weekly calls and instead are called monthly. If during these monthly calls, the participant is reached, they are returned to the weekly call list.

### Survey Delivery and Data Collection

The data collection team is comprised of 16 interviewers who report to 2 supervisors and 1 call center manager. Questionnaire data are collected on password-encrypted tablets using SurveyCTO software. The software presents a list, unique to each interviewer, of participant names and phone numbers. Independent of the survey software, the interviewer tracks which participants are called that day. A participant may need to be called multiple times during a shift due to not picking up the call or asking to be called back. The interviewer only uploads 1 form per contacted participant per shift to SurveyCTO. All survey data and voice recordings are stored in the device memory and then submitted to the SurveyCTO server whenever transmission is possible (internet connection needed), preferably every day but at minimum once a week to minimize risk of data loss. The same tablet is used to call participants and record data.

### Incentives

Participants are compensated for their time with a small amount of phone credit. Studies have shown that in 2 countries (Uganda and Bangladesh), an airtime incentive improved response rates [35]. The World Bank ran a 6-country study in Africa, during which participants were called monthly. Participants received incentives during that study, and response rates were greater than 90% in all countries [6,15]. For this study, the incentive is sent as phone credit, and there is no handling of cash or in-person disbursement of compensation. All participants were given 35 maloti (US $2) by LeCellPHIA administrative staff within 2 weeks of enrolling. Thereafter, participants are given a monthly incentive dependent on how many calls they participated in that month. Between the months of July and December, the incentives were sent by mobile phone companies in Lesotho. However, the administrative process of transferring the money and phone numbers to the mobile phone companies and then the companies sending the incentive was not completed in a timely matter. Due to these delays in phone credit disbursement, which were noted by survey participants, a LeCellPHIA staff began distributing the incentive in-house.

### Supervision and Monitoring

SurveyCTO audio records a preprogrammed percentage of randomly selected interviews and uploads them to the server. Recordings begin at the start of selected calls. Interviewer monitoring was more intensive at the beginning of data collection. Supervisors targeted interviewers who underperformed as compared to their peers. Indicators to measure interviewer performance include response rates and efficiency. During the first month of data collection, supervisors met virtually with interviewers multiple times a week to share performance feedback. From month 2 of data collection onward, supervisors conduct one-on-one sessions biweekly with interviewers to check on their well-being, listen to their concerns, and boost their morale.

### Institutional Review Board Approval, Ethics, Consent

The Lesotho National Research Ethics Committee approved LeCellPHIA with exemption from committee review. It was determined to be a continuation of LePHIA2020, which had already been approved, and the Columbia University Institutional Review Board (IRB) determined the same. The CDC International Task Force scientific committee and the CDC IRB reviewed the protocol and deemed the research nonhuman subjects. Due to the remote nature of data collection (ie, no in-person interaction) and the minimal risk to participants, we requested a modification to written signed informed consent. Participants were read an abbreviated standardized verbal disclosure (consent) of key study information that emphasized the voluntary nature of the survey.

### Statistical Analysis

#### Power Calculation

We calculated the margin of error for a 95% CI to estimate the proportion of ILI through self-reported symptoms. An effective sample size of 3420 produces a 2-sided 95% CI with a margin of error of 1.4% when the proportion of ILI is 10% and the design effect is 1.3 given a loss-to-follow-up rate of 30%. The potential loss in efficiency due to cluster sampling is minimal in this study due to the large number of PSUs and a small number of persons in each PSU. We used a design effect of 1.3 to primarily reflect the impact of sample weights**.** We oversampled older individuals, as cell phone ownership rate is lower and ILI rate is higher in this population.

#### Data Management and Analysis

The data team developed a cleaning plan that included processes for screening data for duplication, transcription errors, measurement errors, internal consistency, out of range and invalid values, and outliers.

We create weekly point estimates of ILI by downloading the survey data from the SurveyCTO platform, which are then cleaned for analysis. Because participants are often called more than once, we retain the most complete survey for analysis. The data are weighted for unequal probability of selection, nonresponse, and potential undercoverage of the sampling frame. The point estimate of ILI prevalence rate with 95% CI is calculated accounting for the stratified, multistage, cluster sample design and is sent to CDC Lesotho and Lesotho Ministry of Health colleagues, usually 3 days after data collection finished.

A monitoring form is also updated weekly to track interviewer response rates and performance, as well as to provide a breakdown of symptoms and a summary of overall data, for both individual participants and their respective household members.

## Results

Over 99% (11,975/12,086, 99.08%) of phone numbers of LePHIA2020 participants who provided consent for follow-up were valid and thus included in the sample frame. We sampled 3020 LePHIA2020 participants. Interviewers enrolled participants for 2 weeks, beginning July 1, 2020. Ultimately 1778 participants were enrolled. Weekly phone calls enquiring about the participant’s symptoms as well as household members listed during the face-to-face survey began the third week of July 2020 and are scheduled to continue until the end of September 2022.

## Discussion

The COVID-19 pandemic caused the already rapidly advancing field of remote data collection in SSA to evolve even faster [36], as collecting data via cell phones was the safest option during periods of high community transmission and national lockdowns. A similar influx of cell phone–based approaches was seen after the Ebola outbreak of 2014-2015 in West Africa, when surveillance via cell phone became more prominent, particularly employing health care staff, such as those working in a health facility or community health workers, to report data [37-41]. Compared with facility-based approaches, participatory surveillance in SSA is less frequent. This protocol manuscript presents the approach used in Lesotho to create weekly estimates of ILI.

There are limitations to cell phone–based data collection that should be considered before establishing a participatory surveillance system. If cell phone ownership is below 80%, undercoverage will occur and could cause coverage bias, impacting the outcome of interest [42]. Specifically, if those who own a cell phone (and thus are part of the sample frame) are different from those without a cell phone in ways that are correlated to the outcome of interest, the estimates will be biased [43]. If mobile phone network coverage is limited mainly to urban areas, then the systematic exclusion of those in rural areas could also create bias in the outcome of interest. Thus, we recommend carefully examining mobile phone ownership in the study setting before attempting participatory surveillance in LMIC. If participants may be hesitant to report the outcome of interest, due to social desirability bias or fear of reporting repercussions, the interviewers must work hard to establish rapport and avoid measurement error.

The usefulness of cell phone–based surveillance systems in resource-limited settings for epidemic response was established by previous studies [44]. Our study built on this work and created population-based, nationally representative estimates of ILI. Whereas previous studies mainly created counts of an outcome, LeCellPHIA creates incidence point estimates with 95% CIs. Because many COVID-19 cases are asymptomatic, the surveillance system may underreport total cases but nonetheless reflects ILI trends across the country.

A surveillance system that can track epidemic trends has the potential to create a more effective response to, in this case, COVID-19. By surveying lay people, we create data in contexts where community health workers or health facility staff are occupied with other tasks. LeCellPHIA can be used as a blueprint to create other population-based cell phone surveillance systems for future outbreaks. 

## Abbreviations

CATI: computer-assisted telephone interview
CDC: Centers for Disease Control and Prevention
HH: household
ICC: intraclass correlation
ILI: influenza-like illness
IRB: Institutional Review Board
IVR: interactive voice response
LeCellPHIA: Lesotho Cell Phone PHIA
LePHIA2020: 2020 Lesotho Population-Based HIV Impact Assessment
LMIC: low- and middle-income country
PHIA: Population-based HIV Impact Assessment
PSU: primary sampling unit
RDD: random digit dial
SSA: sub-Saharan Africa
